# Modified Bufei decoction improves calf health by enhancing immunity, reducing inflammation, and modulating gut microbiota

**DOI:** 10.3389/fvets.2025.1689481

**Published:** 2025-10-29

**Authors:** Zhuo Yang, Peng Ji, Yongli Hua, Chenchen Li, Haochi Yang, Fanlin Wu, Xiuqin Wang, Yanming Wei, Jianpeng Yan

**Affiliations:** ^1^College of Veterinary Medicine, Gansu Agricultural University, Lanzhou, Gansu, China; ^2^Lanzhou Institute of Husbandry and Pharmaceutical Science, Chinese Academy of Agricultural Sciences, Lanzhou, Gansu, China; ^3^Institute of Animal Science, Ningxia Academy of Agriculture and Forestry Sciences, Yinchuan, Ningxia, China

**Keywords:** modified Bu-Fei decoction, calf pneumonia, network pharmacology, molecular docking, 16S rDNA

## Abstract

**Objective:**

To analyze the active ingredients of Modified Bufei Decoction (MBFD) and their targets of action, as well as to validate the preventive effects of its modified formulation on respiratory diseases in calves.

**Methods:**

Network pharmacology and molecular docking were employed to identify active components and targets. Sixty calves were randomly allocated to control and MBFD groups (21-day intervention). Pneumonia incidence, serum inflammatory factors, immune parameters, and antioxidant capacity were measured, with gut microbiota analyzed by 16S rDNA sequencing. The Spearman method was used to analyze the correlation between the differential intestinal flora and the total ingredients.

**Results:**

Systematic screening identified 233 bioactive components in MBFD that target key molecules, such as TNF and IL-6, and modulate pathways, including IL-17 and PI3K/AKT. Molecular docking suggests preliminary binding indications between them. There was a 17% reduction in pneumonia incidence with MBFD (43.3%) compared to the control group (60%). Immunoglobulins (IgA, IgG, and IgM) and antioxidants (CAT, GSH, T-SOD, and T-AOC, all with *p* < 0.05) showed an increasing trend, while inflammatory cytokines (IL-6, TNF-*α*, and IL-1β) and MDA exhibited a decreasing trend. The predominant phyla identified through 16S rDNA sequencing were *Firmicutes*, *Bacteroidetes*, and *Proteobacteria*. After drug administration, the relative abundance of *Bacteroidetes* increased compared to the control group.

**Conclusion:**

MBFD enhances growth, immunity, and intestinal health in calves by modulating the gut microbiota, alleviating dysbiosis, and reducing the incidence of respiratory diseases. This study provides a basis for the rational application of MBFD in calf healthcare.

## Graphical abstract

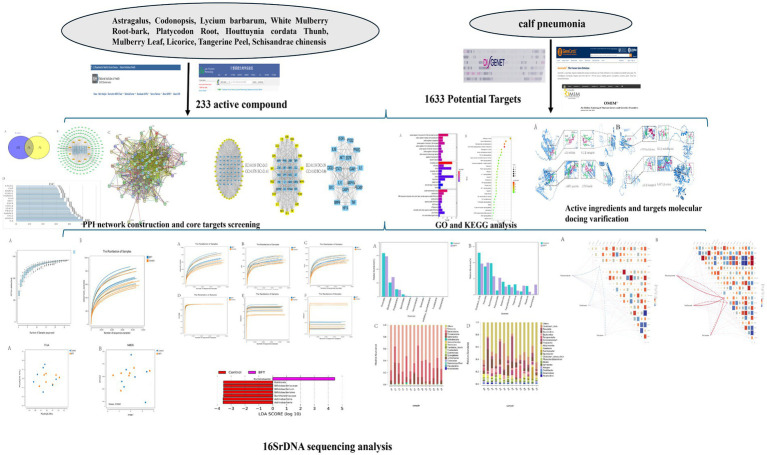

## 1 Introduction

Infectious calf pneumonia, a type with a high mortality rate in confined cattle, is a relatively common and frequently occurring respiratory disease in calves (1, 37) and often jeopardizes calf health (41). Some researchers found that the etiology of calf pneumonia was complex, involving the influence of environmental conditions, feeding management practices, and blood-borne infections (2, 38). Clinical manifestations of pneumonia include shortness of breath, abnormal lung sounds on auscultation, respiratory distress, depression, and decreased resistance (39). Once calf pneumonia has begun, the disease progresses slowly and is susceptible to secondary infections. This significantly increases treatment time and cost, resulting in economic losses for farmers. Therefore, large-scale farmers should pay great attention to strengthening epidemic prevention and disease control. The incidence and severity of calf pneumonia are closely related to the management and disease patterns of individual farms, so the optimal approach is to implement a specialized program for each farm (3). Previous studies have indicated that penicillin-procaine injection powder and tilmicosin may be effective in treating calf pneumonia. However, as antibiotics, they carry side effects, such as the development of drug resistance (4, 5). The clinical treatment of infectious calf pneumonia is mainly based on Western medicine. However, literature has reported that combining it with Traditional Chinese Medicine (TCM) can achieve better therapeutic results (6). Therefore, there is an urgent need to screen Chinese herbal compounds and active ingredients from TCM to treat calf pneumonia.

The Bufei decoction originated from the “Inscription Formula of Yong Class” by Li Zhongnan in the Yuan Dynasty. Due to the high cost of herbs and other problems, the original formula was modified, and the Modified Bu-Fei Decoction (MBFD) was derived from the Bu-Fei Decoction. This formula consists of 10 herbs, including *Astragalus*, *Codonopsis*, *Lycium barbarum*, *Schisandra chinensis*, *White Mulberry Root-bark*, *Mulberry Leaf*, *Houttuynia cordata Thunb*, *Platycodon Root*, *Licorice*, *and Tangerine Peel*. This formula is commonly used in clinical practice to treat lung deficiency diseases, tonify the lungs, astringe the lungs, and clear the lungs (7, 8). In this formula, *Codonopsis* and *Astragalus* membranaceus are the principal herbs. Both are beneficial for tonifying qi and are used in combination to reinforce Lung-qi (9); supplemented with *Schisandrae chinensis* to astringe the Lung-qi, tonify the kidney, and promote the production of body fluids (10); with *Mulberry Leaf* and *Houttuynia cordata Thunb* to clear lung heat and dissolve phlegm in the lungs; and with *Platycodon Root*, *Licorice* and *Tangerine Peel* to eliminate phlegm and relieve cough, lower qi and calm asthma ((11–14)).

Chinese medicine is distinguished by its incorporation of multiple components, targets, and pathways. Due to the complex composition of MBFD and its role in preventing and treating calf pneumonia, the key active ingredients and the underlying mechanisms remain unclear. Network pharmacology is a common approach to studying TCM, whereby the “drug-component-target” network relationship is established to discover new drug targets and elucidate TCM’s material basis and mechanism of action (15, 16). Recent advances in bioinformatics have made research more multidisciplinary. This has led to the widespread use of network pharmacology and 16S rDNA sequencing in the study of TCM, including both single herbs and formulas. Network pharmacology provides a fundamental framework and overarching concept for treating TCM. 16S rDNA sequencing is used to study intestinal microbial diversity. It is a valuable method for elucidating TCM’s mechanisms of action. It also helps in ensuring the quality of Chinese herbal medicines.

This study used a combination of network pharmacology and 16S rDNA sequencing to investigate how MBFD prevents calf pneumonia. Additionally, molecular docking technology was used to validate the binding interactions between the main pharmacodynamic components and their targets. Serum biomarker analysis revealed that MBFD enhances immunity and antioxidant capacity in calves, modulates the composition of the gut microbiota, and improves growth performance. These findings lay the groundwork for further research into the potential mechanisms of MBFD in preventing calf pneumonia and for a more comprehensive investigation of its active components.

## 2 Materials and methods

### 2.1 Materials and reagents

The Animal Ethics Committee at the Gansu Agricultural University (Lanzhou, China) approved all animal experiments in this study under the approval number (GSAU-Eth-VMC-2022-316).

We used enzyme-linked immunosorbent assay (ELISA) kits (Shanghai Enzyme-linked Biological Co., Ltd., China) to measure serum levels of the inflammatory factors interleukin-6 (IL-6, ml064296), interleukin-1β (IL-1β, ml064295), and tumor necrosis factor-*α* (TNF-α, ml077389) in calves. Additionally, the biochemical kits (Nanjing Jiancheng Bioengineering Institute) were used to measure the following activities: total antioxidant capacity (T-AOC, A015-2-1), total superoxide dismutase (T-SOD, A001-1-2), malondialdehyde (MDA, A003-2-2), catalase (CAT, A007-1-1), and reduced glutathione (GSH, A006-2-1).

### 2.2 Preparation of MBFD

According to TCM’s standard decoction preparation method, 100 g of compound conventional Chinese medicine (including *Astragalus*, *Codonopsis*, *Lycium barbarum*, *Schisandra chinensis*, *White Mulberry Root-bark*, *Mulberry Leaf, Houttuynia cordata Thunb*, *Platycodon Root*, *Licorice*, and *Tangerine Peel*). These ingredients were procured from the Yellow River Herb Market in Lanzhou, China. The samples were then weighed and pulverized into a fine powder. Then, 10 times the quantity of water was added to the mixture, which was allowed to soak for 1 h. Finally, the solution was filtered through four layers of gauze. The sediment precipitated with the dregs. Then, add eight times the amount of water, decoct for 40 min, filter, and combine the two filtrates. Centrifuge at 3,000 rpm for 5 min, collect the supernatant, concentrate, and dry to obtain the test drug (yield: 20.64%). Finally, carry out low-temperature freeze-drying and preservation.

### 2.3 Component identification analysis of MBFD

A certain quantity of MBFD extract was weighed and dissolved in deionized water, 60% ethanol, and methanol to prepare test solutions with concentrations of 0.5, 10, and 20 mg/mL, respectively. Each solution was ultrasonicated, fully dissolved, filtered through a 0.22 μm membrane, and then used for the determination of total polysaccharides, total flavonoids, and total saponins.

#### 2.3.1 Determination of total polysaccharides, total flavonoids, and total saponins

Total polysaccharides, total flavonoids, and total saponins were determined according to the literature improvement (17), briefly as follows:

Total polysaccharides: 0.4 mL of MBFD’s aqueous extract was mixed with 0.2 mL of a 5% phenol solution and 1 mL of sulfuric acid. The reaction was conducted at 40 °C for 15 min and then cooled under running water. Then, 200 μL of the sample solution was pipetted into a 96-well plate and the OD value was measured at 490 nm. The total polysaccharide content of the MBFD sample was then converted according to the standard curve.

Total flavonoids: 0.5 mL of aqueous extract of MBFD was mixed with 30 μL of 10% sodium nitrite solution and reacted for 6 min at room temperature, then 30 μL of 10% aluminum nitrate solution was added and reacted for 6 min, then 0.4 mL of 10% sodium hydroxide solution was added and mixed well. The solution was then diluted to 1 mL with deionized water, and 200 μL was transferred to a 96-well plate to measure the OD at 510 nm for calculation against the standard curve. The OD value was determined, and the total flavonoid content was converted from the standard curve.

Total saponins: Take the appropriate amount of aqueous extract of MBFD in a test tube, fully evaporate, and dry at 60 °C. Add 100 μL of 8% vanillin ethanol solution and 1 mL of 72% sulfuric acid, mix well, and then react accurately at 60 °C for 10 min. Remove the running water and cool. Then, 200 μL of the sample solution was pipetted into a 96-well plate and the OD value was measured at 490 nm. The total saponin content of the MBFD sample was then converted according to the standard curve.

#### 2.3.2 Linear relationship

Preparation of standard curve for glucose standard: Pipette 0.2 mg/mL glucose standard solution 0, 20, 40, 60, 80, 120, 160 μL, respectively, according to the above method of total polysaccharide determination, and determine the absorbance OD value. Plot a standard curve for glucose with the concentration of the reference solution on the x-axis and the absorbance on the y-axis to obtain the linear regression equation.

Preparation of standard curve for rutin standard: Pipette 0.1 mg/mL rutin standard solution 0, 50, 100, 200, 300, 400, and 500 μL, respectively, according to the above method for the determination of total flavonoids, and determine the absorbance OD value. Plot a standard curve for rutin with the concentration of the reference solution on the *x*-axis and the absorbance on the y-axis to obtain the linear regression equation.

Preparation of ginsenoside Rb1 standard curve: 0.5 mg/mL ginsenoside Rb1 standard solution, 0, 40, 60, 80, 100, 120, 130, and 140 μL were taken up, respectively, and the absorbance OD value was measured according to the above method for the determination of total saponins. Plot a standard curve for ginsenoside Rb1 with the concentration of the reference solution on the x-axis and the absorbance on the y-axis to obtain the linear regression equation.

### 2.4 Network pharmacology

#### 2.4.1 Mining and screening of pharmaceutical ingredients in MBFD

The principal chemical constituents of *Astragalus*, *Codonopsis*, *Lycium barbarum*, and other related species were extracted from the TCMSP database. The active ingredients of each drug were screened one by one in the TCMSP database^1^ on the principle of Oral Bioavailability (OB) ≥ 30% and Drug Likeness (DL) ≥ 0.18.

#### 2.4.2 Collection of drug targets and gene name conversion in MBFD

The corresponding action targets of the aforesaid active ingredients were gathered in the TCMSP database, and the targets of each drug were integrated by employing the Strawberry Perl software^2^. Moreover, the components of all the drugs obtained above were transformed into Gene symbols in combination with the Uniprot database^3^; that is, the corresponding gene names were obtained, which were utilized for the subsequent network analysis.

#### 2.4.3 Acquisition of calf pneumonia-related targets

Using GeneCards^4^, OMIM^5^, and DisGeNET databases^6^, enter “calf pneumonia” to search for disease targets related to calf pneumonia, sum up all the targets obtained, and eliminate duplicates to obtain the total disease targets.

#### 2.4.4 Take the intersection of “drug target-disease target” to obtain the core target

Using the Venn 2.1 drawing tool to take the intersection of drug and disease targets to generate a Venn diagram, that is, to get the common target of the treatment of calf pneumonia with MBFD.

#### 2.4.5 Construct “drug-component-target” network diagrams

The previously identified intersected gene targets were imported into Cytoscape 3.9.1 to generate a visualized “drug-component-target” network. Core bioactive components were subsequently analyzed using the CytoNCA plugin.

#### 2.4.6 Construction of PPI network

The intersections obtained from the Venn analysis were imported into the STRING database^7^. The species was limited to “Bovine,” the interaction threshold was set to 0.4, the free points were hidden, and the high-definition PPI network diagram and TSV file were exported. The TSV file was imported into the Cytoscape 3.9.1 software for the topological analysis. The core targets were filtered according to the betweenness centrality (BC), closeness centrality (CC), degree centrality (DC), and eigenvector centrality (EC).

#### 2.4.7 Pathway enrichment analysis

The DAVID database^8^ was used to analyze the potential target genes of MBFD. Gene Ontology (GO) enrichment analysis (*p* < 0.05) and Kyoto Encyclopedia of Genes and Genomes (KEGG) pathway enrichment analysis (*p* < 0.05) were used to analyze the biological process (BP), cellular component (CC), and molecular function (MF). These analyses revealed significant biological pathways of MBFD’s active ingredients against calf pneumonia.

#### 2.4.8 Molecular docking of components and targets

Molecular docking was performed on the top eight core components and the top five core targets obtained from the PPI network analysis. Structure diagrams of the small molecules were acquired from the TCMSP database and downloaded in mol2 format. The optimal structures of the five core target proteins were obtained from the UniProt and PDB databases^9^ and downloaded in PDB format. The proteins were subjected to dehydrogenation and water addition utilizing the Autodock Tools software. The binding energy < 0 indicates that the ligand and receptor can bind spontaneously, and the binding energy ≤ − 5.0 kJ/mol indicates that the molecules dock well with the targets. The docking results were presented by the PyMOL software.

### 2.5 Animal experiments

#### 2.5.1 Test animals

Sixty calves weighing (122.55 ± 15.01 kg) at the age of 3 months were selected and randomly divided into control and MBFD groups, with 30 calves in each group. The experiment was conducted at Wanhe Grass and Livestock Industry Science and Technology Development Limited Liability Company’s cattle farm in Zhangye City, Gansu Province, China. The calves in the control group were fed a basal ration daily. Those in the MBFD group received a lung tonic soup containing 15 g/kg of concentrate daily, in addition to the basal ration, for 21 days. All calves were fed ad libitum once daily and had free access to feed and water.

#### 2.5.2 Sample collection

At the conclusion of the experiment, seven healthy calves were selected from the control group and seven affected calves were selected from the MBFD group. Approximately 5 g of fresh feces were collected from each calf and stored in a freezer at −80 °C. Blood samples were simultaneously collected from each calf into non-anticoagulant tubes. After coagulation, the samples were centrifuged to obtain the serum from the supernatant. The serum was divided into smaller portions, or aliquots, and stored at −20 °C.

#### 2.5.3 Morbidity rate

Daily observation of the health status of each group of calves shall be conducted, with detailed records kept of clinical symptoms such as coughing, ocular discharge and nasal discharge. An analysis of the incidence rates between different calf groups shall be conducted. The incidence rate is calculated as follows: Incidence rate = (Number of affected heads in each group / Total number of heads in each group) × 100%.

#### 2.5.4 Measurement of serum biochemical indicators

Serum levels of IL-6, TNF-*α*, IL-1β, T-SOD, MDA, CAT, T-AOC, GSH, IgA, IgM, and IgG were analyzed according to the kit instructions.

### 2.6 High-throughput sequencing of intestinal flora

#### 2.6.1 16S rDNA sequencing and flora analysis

The PCR reaction system was set up with 30 ng of high-quality genomic DNA samples and the corresponding fusion primers, and the PCR parameters were optimized for PCR amplification. The PCR amplification products were purified by using Agencourt AMPure XP magnetic beads, dissolved in an Elution Buffer, labeled, and then built into the library. The fragment range and concentration of the libraries were analyzed with the help of the Agilent 2,100 Bioanalyzer. The qualified libraries were sequenced according to the size of the insert fragments. For downstream analysis, the remaining high-quality clean data were prepared. Subsequently, reads were spliced into tags based on overlapping regions. These tags were then clustered into OTUs. The OTUs were compared against a database for taxonomic annotation. Based on the OTU and annotation results, species complexity within samples was analyzed. Additionally, differences in species composition between groups were examined. All sequencing and bioinformatics services were provided by Wuhan Welltec Genetics Co.

### 2.7 Correlation analysis

Perform correlation analysis between the total components and 16S rDNA results using the Spearman method.

### 2.8 Data processing

Data were expressed as ‘mean ± SD’ and analyzed statistically using GraphPad Prism9, with *p* < 0.05 being considered significant.

## 3 Results

### 3.1 Content of active ingredients

**Figure 1:**
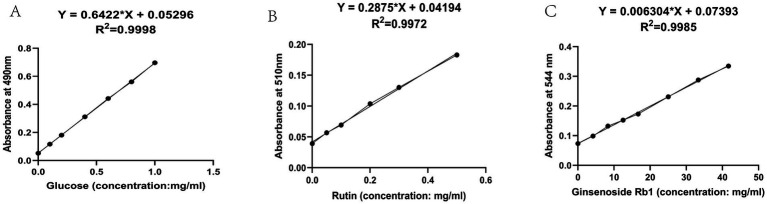
Standard curve diagram. **(A)** Standard curve for polysaccharides **(B)** Standard curve for flavonoids **(C)** Standard curve for saponins.

The linear regression equations and standard curves of each standard were obtained according to the linear relationship investigation, as shown in Table 1 and Figure 1 below. Results indicate that glucose exhibits good linearity between 0–1 mg/mL, rutin demonstrates good linearity between 0–0.5 mg/mL, and ginsenoside Rb1 shows good linearity between 0–50 mg/L. The linear regression equation established as described above yielded 28.29% of total polysaccharides, 0.6442% of total flavonoids, and 5.79% of total saponins.

**Table 1:** Linear regression equations.

Standard product	Linear regression equation	*R* ^2^	Linear range
Total polysaccharides (glucose)	y = 0.6422*x + 0.053	0.9998	0 ~ 1 mg/mL
Total flavonoids (rutin)	y = 0.2875*x + 0.0419	0.9972	0 ~ 0.5 mg/mL
Total saponins (ginsenoside Rb1)	y = 0.006304*x + 0.0739	0.9985	0 ~ 50 mg/mL

### 3.2 Network pharmacology and molecular docking results

#### 3.2.1 Screening of active ingredients of MBFD

In the TCMSP database, OB ≥ 30% and DL ≥ 0.18 were taken as the filtering conditions to screen out 266 active ingredients of MBFD. The obtained target information was removed from the duplicate values. The integration resulted in 184 targets corresponding to the active ingredients. The active ingredients were obtained as 20 for *Astragalus*, 21 for *Codonopsis*, 45 for *Lycium barbarum*, 7 for *Platycodon Root*, 5 for *Tangerine Peel*, 29 for *White Mulberry Root-bark*, 92 for *Licorice*, 7 for *Houttuynia cordata Thunb*, 32 for *Mulberry Leaf*, and 8 for *Schisandra chinensis*. The active ingredients of each Chinese medicine were combined and organized to eliminate duplicates, and ultimately, 233 active compounds were obtained.

#### 3.2.2 Acquisition and Venn analysis of calf pneumonia-related targets

**Figure 2:**
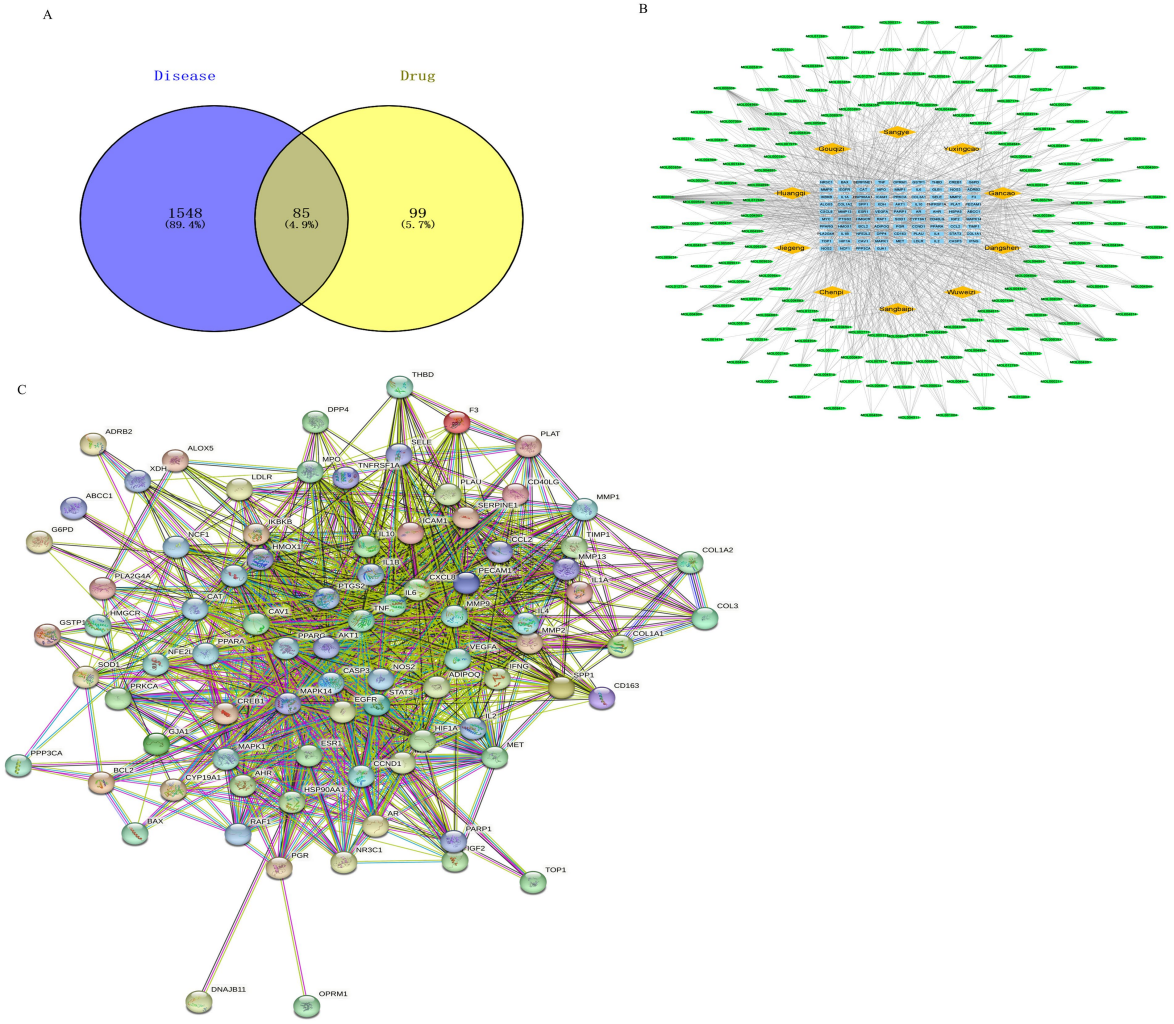
Drug target and disease target diagram and drug-component-target network diagram. Note: Network pharmacology was used to screen the relationship and mechanism of action of MBFD with common core targets of calf pneumonia. **(A)** Venn diagrams obtained 85 cross-targets for MBFD and calf pneumonia **(B)** Analysis was performed using the plug-in CytoNCA, and the top 30 PPI core targets (Yellow diamonds represent drugs, green ellipses represent ingredients, and blue squares represent drug-disease intersection targets) **(C)** Cytoscape 3.9.1 software was utilized to construct the PPI network, and the target sites were ranked according to the number of nodes and edges.

The GeneCards, OMIM, and DisGeNET databases were searched using the keyword “calf pneumonia.” The results were combined, yielding 1,633 disease targets after duplicates were removed. These targets were analyzed using the Venn online mapping tool, yielding a total of 85 intersecting targets (Figure 2A).

#### 3.2.3 Construction of “drug-component-target” network diagrams

To further clarify the potential targets and protein interactions, the protein interaction data, active ingredients, and predicted targets were imported into Cytoscape 3.9.1. A “drug-ingredient-target” network was then constructed, as shown in Figure 2B. The analysis of CytoNCA revealed that the top 10 active ingredients of MBFD were the most important ones, including quercetin (degree = 64), luteolin (degree = 29), kaempferol (degree = 27), naringenin (degree = 19), nobiletin (degree = 15), arachidonic acid (degree = 14), *β*-carotene (degree = 13), licochalcone-A (degree = 13), 7-methoxy-2-methyl isoflavone (degree = 13), formononetin (degree = 12).

#### 3.2.4 Construction and analysis of the PPI network diagram

**Figure 3:**
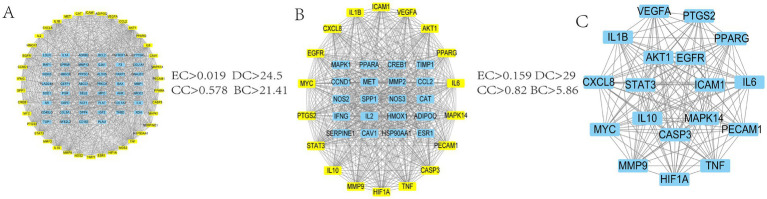
Screening process diagram. **(A)** PPI network obtained by intersecting genes of the active ingredient of MBFD and calf pneumonia **(B)** PPI network filtered from A according to the median criterion **(C)** Final PPI network filtered from B according to the median criterion.

The 85 intersecting targets obtained through Venn’s analysis were imported into the STRING database, with the species being limited to “Bovine.” The resulting protein interaction network was constructed, as shown in Figure 2C. This network had 85 nodes, generated 1,162 edges, and had an average node degree value of 27.3. After a series of screenings, 18 key target genes were ultimately identified. The screening process is presented in Figure 3. The highest correlation values were for TNF, IL-6, serine and threonine kinase (AKT1), vascular endothelial growth factor A (VEGFA), and IL-1β. These genes may play important roles in preventing and treating calf pneumonia with the MBFD and may be core targets of its potential effects.

#### 3.2.5 GO and KEGG pathway enrichment analysis

**Figure 4:**
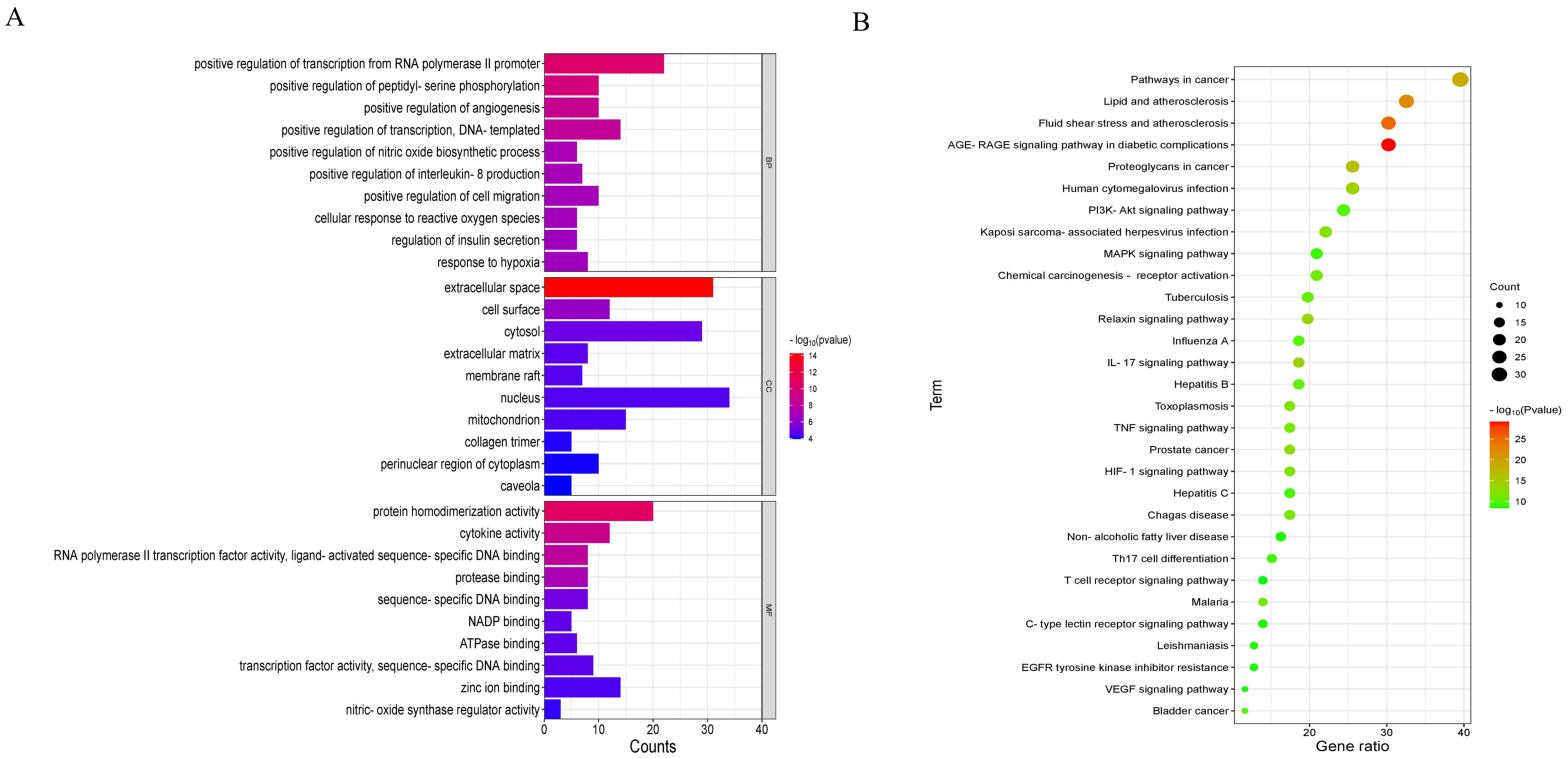
GO and KEGG enrichment analysis (top 30). **(A)** GO analysis of the apoptosis-related core targets showed the top 10 items on IL-17, PI3K-Akt, MAPK, and other signaling pathways. **(B)** KEGG pathway analysis of the core targets showed the top 10 items of acacetin’s anti-hepatic fibrosis activity.

The 85 potential targets of MBFD were analyzed employing the DAVID database. GO enrichment analysis yielded 387 GO entries, and the top 10 rankings of each component are presented in Figure 4A. The KEGG pathway was enriched in 130 signaling pathways, and the top 30 pathways were visualized using bubble diagrams (as shown in Figure 4B). As can be seen in the figure, MBFD is involved in IL-17, PI3K/AKT, MAPK, and other signaling pathways.

#### 3.2.6 Molecular docking validation

**Figure 5:**
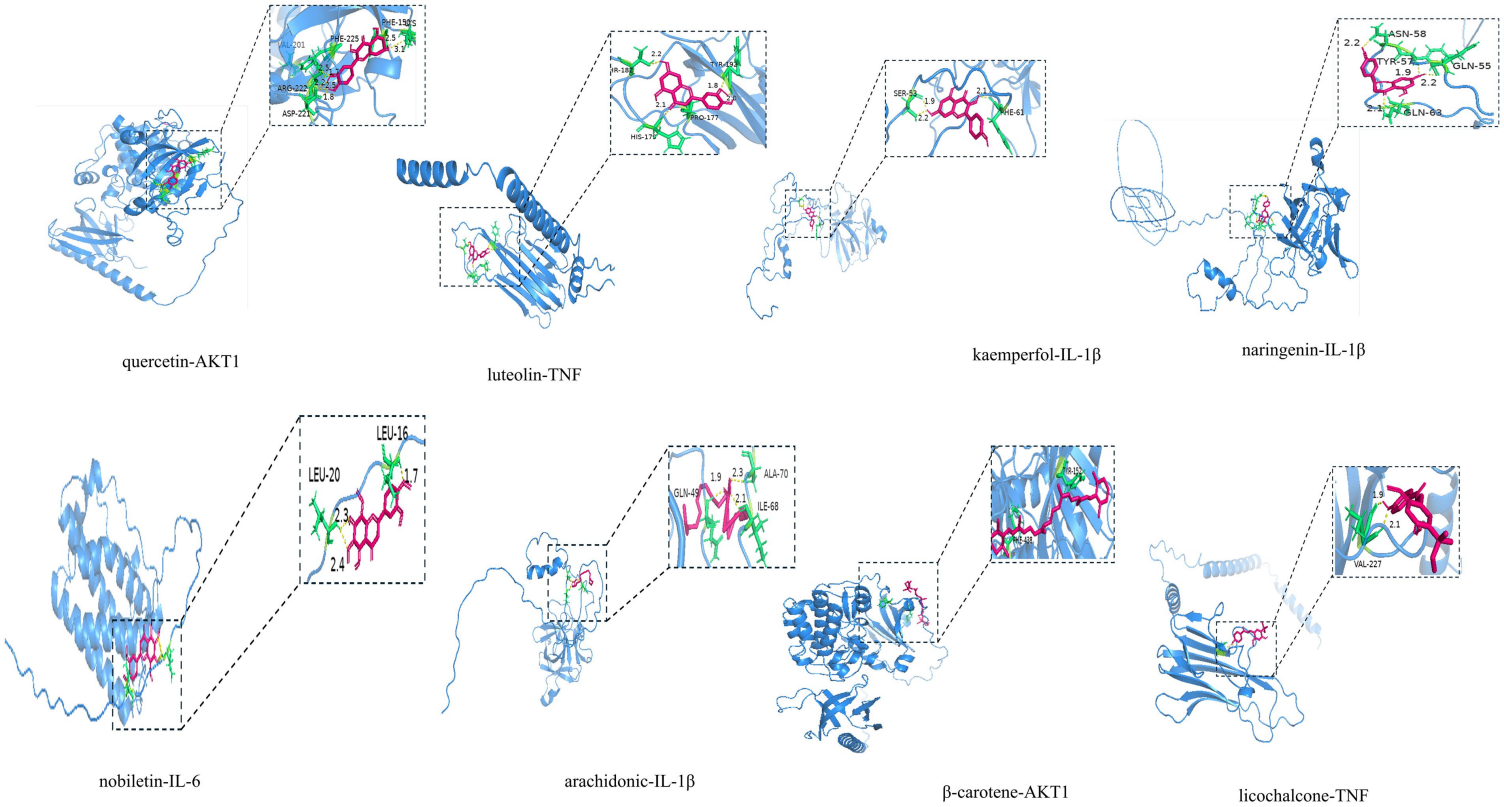
Molecular docking pattern diagram.

To verify the reliability of network pharmacology, the active ingredients of MBFD with the top 8 Degrees (Degree ≥ 13) were used as ligands, and molecular docking was performed with the top 5 core targets as receptors. The docking results are presented in Table 2. Generally, the lower the binding energy between the ligand and the receptor, the greater the docking activity and the more stable the structure. The docking results showed that the binding energies of all the macromolecules and targets were negative, suggesting preliminary binding indications between them. Some visualization results are displayed in Figure 5.

**Table 2:** Molecular docking results.

Serial Number	Compound name	TNF	AKT1	IL-6	IL-1*β*	VEGFA
1	Quercetin	−4.69	−5.55	−4.88	−4.64	−4.89
2	Luteolin	−5.33	−4.69	−4.29	−4.9	−5.02
3	Kaempferol	−4.16	−5.8	−4.47	−6.01	−4.1
4	Naringenin	−2.64	−5.09	−4.35	−5.83	−4.95
5	Nobiletin	−4.2	−3.89	−3.95	−2.75	−3.51
6	Arachidonic acid	−2.96	−3.26	−2.95	−3.96	−2.49
7	β-carotene	−5.4	−6.73	−5.26	−6.36	−5.38
8	Licochalcone	−5.57	−2.96	−5.2	−5.51	−5.05

### 3.3 Incidence rate of calf diseases

As shown in Table 3, the clinical trial results demonstrated that the daily weight gain of calves in the MBFD group was significantly higher than that of the control group. The control group had an incidence rate of 60% (18 out of 30), while the MBFD group had an incidence rate of 43.3% (13 out of 30). Table 4 below shows the incidence rates for each group.

**Table 3:** Effect of MBFD on average daily gain in calves.

Average body weight (kg)	Control group			MBFD group		
	Initial body weight	Final body weight	Daily weight gain	Initial body weight	Final body weight	Daily weight gain
	138.58	155.8	1.15	143.13	164.75	1.44

**Table 4:** Incidence rates by period and by group.

Groups	Control group	MBFD group
Incidence rates	60%	43.3%

### 3.4 Effects of serum biochemical indicators

#### 3.4.1 Effect of MBFD on inflammatory factors and immune indicators in Simmental calves

As shown in Table 5, compared with the control group, the MBFD group exhibited significantly higher serum levels of IgG and IgM (*p* < 0.01). In contrast, IgA levels showed an increasing trend (*p* > 0.05). Additionally, the MBFD group demonstrated markedly reduced serum concentrations of IL-6 and TNF-*α* (*p* < 0.01), along with significantly decreased IL-1β levels (*p* < 0.05).

**Table 5:** Effects of feeding MBFD on serum immune factor indices in Simmental calves.

Items	Control group	MBFD group
TNF-α (pg/mL)	78.60 ± 2.13^b^	72.54 ± 1.41^a^
IL-6 (g/L)	5.56 ± 0.47^b^	2.74 ± 0.22^a^
IL-1β (pg/mL)	519.84 ± 16.39^b^	485.78 ± 18.02^a^
IgG (pg/mL)	73.29 ± 4.85^b^	103.59 ± 4.93^a^
IgM (ug/mL)	193.22 ± 18.43^b^	458.56 ± 40.81^a^
IgA (ug/mL)	178.11 ± 17.41^a^	192.93 ± 20.25^a^

#### 3.4.2 Effect of feeding MBFD on serum antioxidant indexes in Simmental calves

As shown in Table 6, Simmental calves fed with MBFD showed significantly increased levels of CAT, GSH, T-SOD, and T-AOC compared to the control group (*p* < 0.05). A decreasing trend in MDA content was observed in the MBFD group compared to the control group (*p* > 0.05).

**Table 6:** Effects of MBFD feeding on serum antioxidant indices in Simmental calves.

Items	Control group	MBFD group
CAT (U/mL)	0.42 ± 0.14^b^	1.65 ± 0.44^a^
GSH (μmol/gprot)	18.47 ± 4.87^b^	57.06 ± 8.38^a^
MDA (nmol/mL)	3.19 ± 0.51^a^	2.55 ± 0.44^a^
T-SOD (U/mL)	115.79 ± 0.52^b^	123.80 ± 1.06^a^
T-AOC (U/mL)	3.38 ± 0.04^b^	3.49 ± 0.02^a^

### 3.5 Results of 16S rDNA sequencing

#### 3.5.1 Sequencing sample size reasonableness assessment

**Figure 6:**
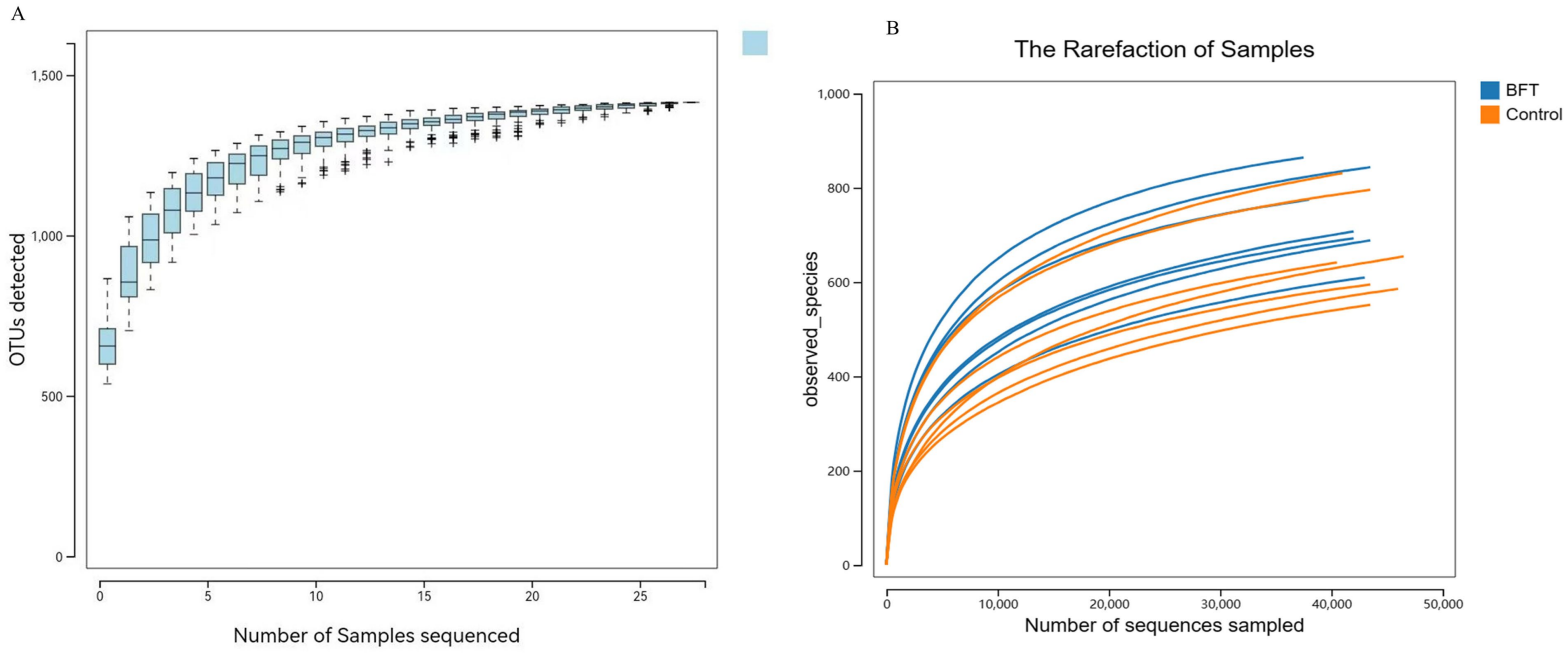
Species accumulation curve and diversity dilution curve. **(A)** The species accumulation curve graph **(B)** The dilution curves.

Species accumulation curves are used to assess the trend in the number of observed species (or OTUs) as the sample size increases. As shown in Figure 6A, the curve gradually flattens with increasing sample size, indicating that the composition of the gut microbiota has stabilized. Even with further increases in sample size, the types of microbial species show no significant changes. Similarly, their abundance also remains largely unchanged. These results demonstrate that the current sample size meets analytical requirements and is suitable for subsequent experiments.

Rarefaction curves evaluate the effect of sequencing depth on species diversity detection. As illustrated in Figure 6B, the curves for all samples gradually plateau with increasing sequencing volume. Rarefaction curves stabilized under different diversity indices. This shows that more data would not significantly increase OTU detection, confirming sufficient sequencing depth and reliable data quality.

#### 3.5.2 Alpha diversity analysis

**Figure 7:**
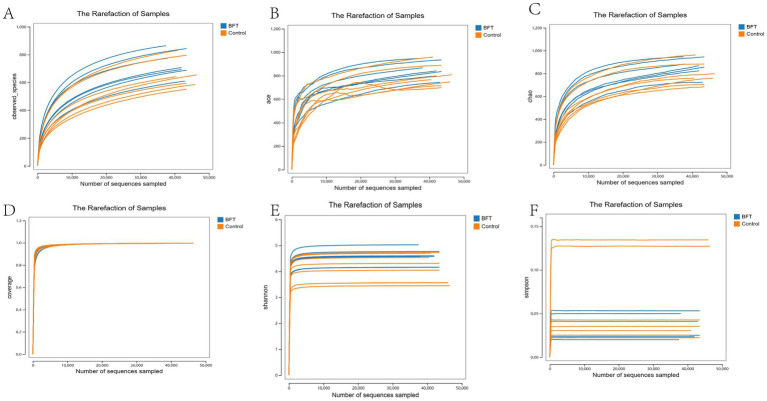
Alpha diversity dilution curve. **(A)** Observed index showed that the BFT group was significantly higher compared to the control group **(B)** The Ace index showed that the BFT group was significantly higher compared to the control group **(C)** Chao1 index showed that the BFT group was significantly higher compared to the control group **(D)** Coverage indices of each group were all greater than 0.99 **(E)** Shannon index showed that the BFT group was significantly higher compared to the control group **(F)** Simpson index showed a significant decrease in the BFT group compared to the control group.

Alpha diversity analysis was used to evaluate the microbial diversity within individual samples, primarily encompassing two dimensions: richness (the number of species) and evenness (the uniformity of species’ relative abundances). This study employed five indices for assessment: Shannon, Simpson, ACE, Chao1, and Observed species. The results showed that the Coverage index for all samples exceeded 0.99, indicating a sequencing coverage rate of over 99% and high data reliability. The rarefaction curve plateaued with increasing sequencing volume, suggesting that the current sequencing depth had sufficiently captured most species present in the samples. As shown in Figure 7, compared to pre-treatment levels, the Simpson index decreased significantly in the MBFD group after treatment, while the Chao1, ACE, and Shannon indices increased significantly.

#### 3.5.3 Principal component analysis

**Figure 8:**
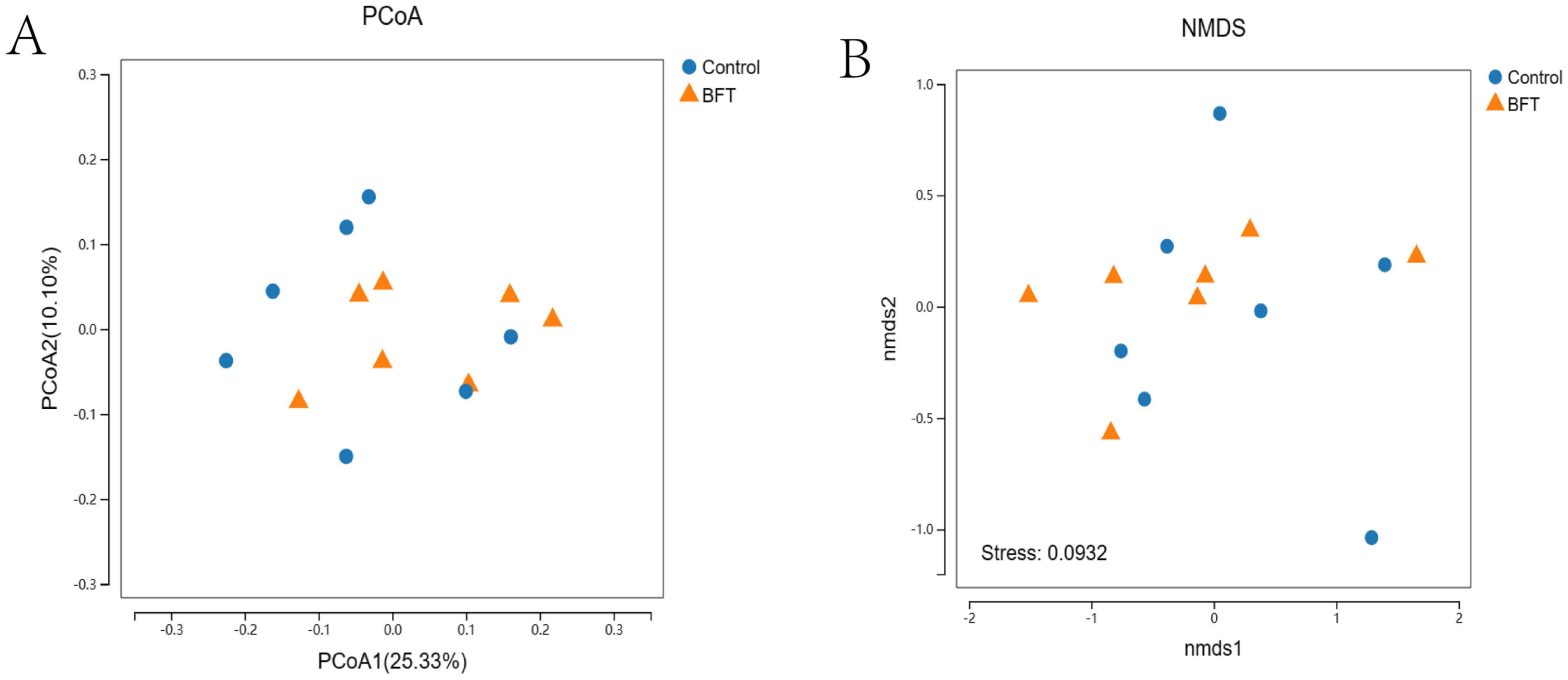
Beta diversity intergroup analysis of variance. **(A)** PCoA shows that the samples are farther apart on the coordinate plot and that there is a difference in the compositional structure of the species **(B)** NMDS shows that the samples are more distant on the coordinate plot and that there is a difference in the compositional structure of the species.

The differences between groups, as shown in the PCoA and NMDS analyses, were displayed in the coordinate plot. In this plot, samples that were closer together had more similar species compositions in terms of *β*-diversity (Figure 8). The contributions of PCoA1 and PCoA2 to the variation among samples were 25.33 and 10.1%, respectively, and the clear separation indicated that the distribution of bacterial species between the two groups was broader and more distinct.

#### 3.5.4 Analysis of species and their abundance

**Figure 9:**
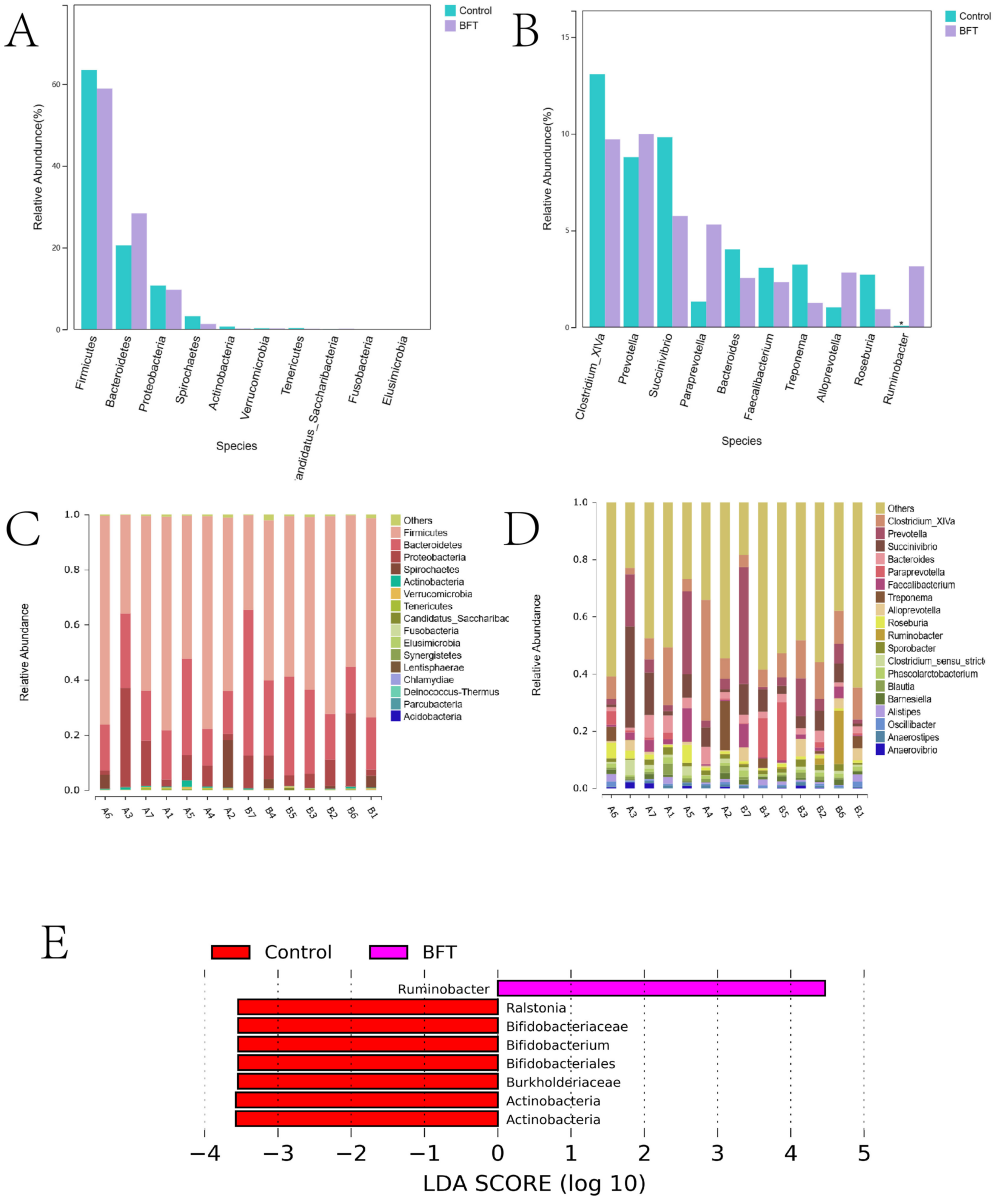
Differences in microbial species distribution at the phylum level and genus level. **(A)** Comparison of key species differences at the phylum level **(B)** Comparison of key species differences at the genus level **(C)** Maps of species composition at the phylum level **(D)** Maps of species composition at the genus level.

Figure 9A shows the top 10 species at the phylum level in terms of relative abundance of bacteria in the calf intestinal samples. Figure 9C shows species composition. The dominant phyla were *Firmicutes*, *Bacteroidetes*, *Proteobacteria*, and *Spirochaetes*. Following administration of the drug, the relative abundance of *Bacteroidetes* increased compared to the control group. The dominant phylum in the MBFD was *Bacteroidetes*, accounting for 28.38 and 20.51% in the MBFD and control group, respectively. Proteobacteria followed, accounting for 9.67 and 10.72% in the MBFD and control group, respectively. Figure 9B shows the top 10 bacterial groups in terms of relative abundance in the calf intestinal samples at the genus level. Figure 9D shows species composition. The dominant genera in the calf intestine were *Clostridium XIVa*, *Paraprevotella*, *Bacteroides*, *Faecalibacterium*, and *Treponema*. In the lung tonic soup group, the dominant genera were *Proteobacteria*, *Paraprevotella*, *Alloprevotella*, and *Ruminobacter*, with relative abundances of 9.99, 5.3, 2.82, and 3.14%, respectively. Notably, *Ruminobacter* was significantly more abundant in the control group than in the MBFD group. These results suggest that MBFD treatment can reverse changes in intestinal microbial structure caused by calf pneumonia during the disease’s pathogenesis.

#### 3.5.5 Results of the analysis of differences between groups of intestinal flora

LEfSe (Linear Discriminant Analysis combined with effect size measurement) analysis was employed to investigate categorical differences between groups and identify significantly different bacterial species after MBFD intervention. The results are presented in Figure 9E. *Ruminobacter* was the dominant phylum in MBFD. In comparison, the dominant bacteria were *Ralstonia*, *Bifidobacteriaceae*, *Bifidobacterium*, *Bifidobacteriales*, *Burkholderiaceae*, and *Actinobacteria*.

#### 3.5.6 Spearman correlation analysis between intestinal flora and total composition

**Figure 10:**
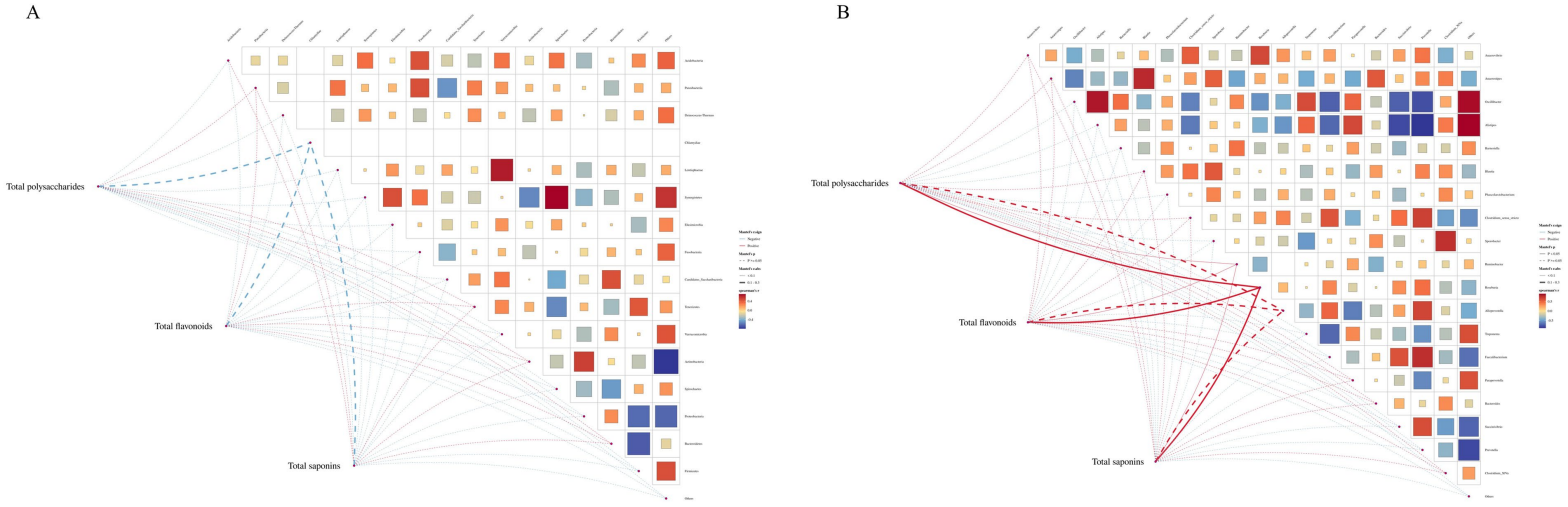
Lefse analysis diagram.Note: Lefse analysis plot showing Ruminobacter as the dominant phylum in MBFD.

The potential relationship between gut flora and total composition was further analyzed by 16S rDNA gene sequencing. Spearman’s correlation analysis was performed for differential flora and total composition (Figure 10). Correlation coefficients between the relative abundance of highly correlated intestinal flora in the MBFD group and total composition were illustrated. The results showed that at the genus level, total polysaccharides, total flavonoids, and total saponins were positively correlated with the dominant genera of the intestinal flora, *Clostridium-XIVa* (*r* = 0.00492), *Paraprevotella* (*r* = 0.0147), and *Bacteroides* (*r* = 0.0939), and with the dominant genera of *Faecalibacterium* (*r* = −0.05468) and *Treponema* (*r* = −0.0187) were negatively correlated. A positive correlation was observed with *Paraprevotella* (*r* = 0.01476), *Alloprevotella* (*r* = 0.1066), and *Ruminobacter* (*r* = 0.01268), which were the dominant genera in the Lung tonic soup group. At the phylum level, total polysaccharides, total flavonoids, and total saponins were positively correlated with the dominant phylum *Bacteroidetes* (*r* = 0.00508) and negatively correlated with *Firmicutes* (*r* = −0.0335), *Proteobacteria* (*r* = −0.0571), and *Spirochaetes* (*r* = −0.018). These results suggest that the improvement of calf pneumonia by the total components of MBFD is closely related to the changes in intestinal flora.

## 4 Discussion

In recent years, the phenomenon of calves having weak resistance has become increasingly prevalent. This is largely due to poor environmental conditions on farms, which lead to infections by pathogenic microorganisms. It has resulted in an elevated incidence of calf pneumonia. This serious pulmonary respiratory disorder is primarily associated with feeding management, methods, and environment, etc. While Western medicine is currently the predominant treatment, a combination of Chinese and Western medicine may yield superior therapeutic outcomes (18, 19). Therefore, we investigated the potential mechanisms and active ingredients of MBFD for treating calf pneumonia from two perspectives. First, we used network pharmacology to identify active ingredients and target proteins, creating a “drug-ingredient-target” network for MBFD. Second, we analyzed the specificity and potential of MBFD in preventing calf pneumonia through molecular docking to guide subsequent *in vivo* experiments. These analysis results provide a framework for future *in vitro* and in vivo studies, optimizing the use of TCM.

By determining the active ingredients in MBFD, this paper found that the total polysaccharide content was the highest, the total saponin content was the second highest, and the total flavonoid content was the lowest. Building a “drug-component-target” network showed that quercetin, lignocerol, kaempferol, naringenin, and chuanchengpiin are possible active ingredients in MBFD. Quercetin, in particular, has been reported to have antioxidant, anti-tumor, anti-inflammatory, antibacterial, and antiviral effects (20). It inhibits fibroblast proliferation and tuberculosis (21) and promotes lung tissue self-repair. Both quercetin and lignocerol treat radiation pneumonia (40). Kaempferol belongs to flavonoids and has antioxidant, anti-tumor, and anti-inflammatory effects (22). Naringenin also belongs to flavonoids and has anti-inflammatory properties that can improve lung function and reduce inflammatory cells (23). *Astragalus, Tangerine peel, Licorice,* and codonopsis tonify the middle and boost qi (24). Quercetin, kaempferol, and naringenin are the key bioactive components responsible for the pharmacological effects of buzhong yiqu decoction (25). Studies have shown that tonifying lung soup is primarily used to treat respiratory diseases, including chronic obstructive pulmonary disease (42), pulmonary fibrosis (26), and lung cancer (27). It is primarily effective in tonifying the lungs, benefiting qi, clearing fire, resolving phlegm, and relieving cough and asthma.

To identify the key genes associated with the active compounds of MBFD in the context of calf pneumonia, a protein–protein interaction (PPI) network was constructed for screening purposes. This approach led to the identification of key target genes, including TNF, IL-6, AKT1, VEGFA, and IL-1β. These genes were then analyzed further. TNF exhibited the highest degree of value and correlation among these genes, suggesting its potential as a key regulator in the observed biological process. These targets are primarily implicated in physiological processes such as inflammation, oxidative stress, immune activation, and apoptosis. Based on this, this study investigated the regulatory effects of MBFD on immune function by measuring serum inflammatory factors and antioxidant indicators in calves. IL-6, IL-1β, and TNF-*α*, as key pro-inflammatory cytokines, play crucial roles in immune response and inflammation regulation (28–30). The experimental results showed that the MBFD group exhibited significantly lower serum levels of IL-6, TNF-α, and IL-1β, along with markedly higher levels of immunoglobulins (IgA, IgG, IgM). Specifically, IgG reflects systemic immune status, IgM promotes early immune response and IgG production, while IgA mediates mucosal immune defense (31, 32). In terms of antioxidant capacity, the MBFD group exhibited significantly elevated activities of CAT, GSH, and T-SOD, as well as increased T-AOC, accompanied by a substantial reduction in MDA levels. This demonstrates that MBFD effectively enhances antioxidant defenses. The comprehensive results suggest that MBFD may improve calf health by suppressing inflammatory responses, enhancing humoral immunity, and alleviating oxidative stress. These synergistic effects collectively promote immune function and provide important support for healthy growth. This study provides scientific evidence supporting the use of MBFD in managing calf health.

The most significant functions identified through GO and KEGG analyses included protein homodimerization activity, cytokine activity, and sequence-specific DNA binding, which is activated by ligand-activated RNA polymerase II transcription factor activity. Major pathways included the IL-17, PI3K/AKT, and MAPK signaling pathways, as well as other pathways related to inflammatory mechanisms. To validate the network pharmacology findings, molecular docking was conducted between the identified active compounds and key targets. The results showed that quercetin, kaempferol, and lignocerol, the core compounds of MBFD, had an enhanced binding affinity with the core targets. Further investigation is warranted to explore the potential roles of quercetin, kaempferol, naringenin, and lignans in modulating the mechanisms underlying calf pneumonia. These results lay the groundwork for future *in vivo* or *in vitro* studies to confirm the potential mechanism of action of MBFD against calf pneumonia.

The gut microbiota is recognized as playing a crucial role in maintaining the health of hosts and ensuring the optimal production performance of livestock and poultry. This study, therefore, employed 16S rDNA technology to analyze the diversity of gut bacterial composition. The stable trends observed in both the species accumulation and dilution curves collectively indicate that the sample size and sequencing depth used in this study were adequate. This approach effectively covered the vast majority of microbial species present in the samples. Such comprehensive coverage supports the reliability of subsequent analyses of microbial community structure and diversity. Changes in alpha diversity indices revealed that the MBFD treatment significantly increased microbial community richness (Chao1 and ACE indices) and overall diversity (Shannon index). Concurrently, the decrease in Simpson’s index reflected improved species evenness. Taken together, these results demonstrate that drug therapy effectively optimized gut microbiota structure and restored microbial community diversity. A coverage index exceeding 0.99 and the saturated trend of the dilution curve further validate the adequacy and reliability of the sequencing data. This provides robust support for the conclusions above. These findings establish a scientific basis for the clinical application of MBFD in regulating the intestinal microbiome. It shows that species richness and bacterial diversity are significantly increased following drug treatment.

The results of the PCoA and NMDS analyses in this study were consistent, indicating that the diversity and abundance of the rectal microbiota of Simmental calves were similar. Intra-group sample clustering reflects a relatively consistent microbial composition among individuals within each group. This consistency under experimental conditions demonstrates good reproducibility. In contrast, the distinct separation between groups suggests that different treatments or groupings have a significant impact on gut microbiota composition. The cumulative contribution rates of PCoA1 and PCoA2 were 35.43%, revealing pronounced intergroup differences and indicating a broad distribution of bacterial species and significant variation between the two groups. Minor differences were indicated by the relative clustering of samples within each group. Significant differences in microbial composition, however, were suggested by the distant separation between groups. These findings confirm that grouping is a key variable driving microbial community variation and provide a basis for further elucidating the relationship between microbial structural changes and experimental interventions. The study identified the phyla *Proteobacteria* and *Bacteroidetes* as dominant in the rumen of Simmental calves, where they are primarily involved in carbohydrate and protein digestion. The study employed the Shannon, Simpson, Chao1, ACE, and Coverage indices to analyze bacterial diversity and abundance in rectal samples from Simmental calves. The results indicated greater diversity and higher abundance within the rectal microbiota. Furthermore, Spearman’s correlation analysis revealed a significant association between improvement in total MBFD components and corresponding alterations in the gut microbiota in calves with pneumonia.

This study confirms that the TCM compound MBFD significantly enhances average daily weight gain in calves, elevates serum immunoglobulin levels (IgG, IgM), and reduces both respiratory disease incidence and pro-inflammatory cytokine levels (IL-6, TNF-*α*, IL-1β). These findings align with the results reported by Wei et al. (33) regarding *Astragalus* extract’s enhancement of calf growth performance, antioxidant capacity, and immunity. Moreover, the present study demonstrates a more pronounced synergistic effect through the compound formulation. Mechanistically, a significant increase in immunoglobulin levels and a marked decrease in pro-inflammatory cytokines were observed. This is consistent with the report by McNeil et al. (34) regarding echinacea’s effects in bovine respiratory diseases. This suggests that plant extracts may exert a conserved immunostimulatory effect across different species. Furthermore, these findings are analogous to Shen et al.’s (35) investigation into the regulatory effects on calf diarrhoea.

We observed a significant increase in the abundance of *Bacteroidetes* within the intestines of calves in the MBFD group. This finding aligns with the conclusions drawn by Ku et al. (36) in their study of the gut microbiome in calves with diarrhoea. Multiple studies collectively suggest that improvements in immune function may be closely associated with the regulation of the gut microbiota. Furthermore, multiple constituents within this compound formula (such as liquorice and dried tangerine peel) may exert synergistic effects. Compared to single-component studies (34), this demonstrates the multi-targeted, holistic regulatory advantages of the TCM compound. The comprehensive enhancement of immune function, coupled with optimized gut microecology, jointly reduces the risk of respiratory disease occurrence. This corroborates the traditional Chinese medical principle that ‘when vital energy resides within, pathogenic factors cannot invade.’ It also highlights the potential application value of TCM in animal health management.

## 5 Conclusion

The study demonstrates that MBFD enhances immune function and antioxidant capacity in calves. Furthermore, it improves serum indices and modulates the relative abundance of Proteobacteria and Bacteroidetes in the gastrointestinal microbiota. Correlation analysis was performed between the gastrointestinal microbiome and overall components. The results revealed that MBFD’s improvement of calf pneumonia is closely related to changes in the gut microbiota. Additionally, network pharmacology and molecular docking analyses indicated that MBFD’s active components, such as quercetin, regulate key molecules including TNF and IL-6. This regulation subsequently influences the IL-17 signaling pathway and other related pathways. This study provides a basis for the rational application of MBFD in calf healthcare.

## Data Availability

The data presented in the study are deposited in the NCBI repository, accession number PRJNA1346235.

## Footnotes

^1^ http://tcmspw.com/tcmsp.php

^2^ https://strawberryperl.com/release-notes/5.32.1.1-64bit.html

^3^ https://www.uniprot.org

^4^ https://www.genecards.org

^5^ https://www.omim.org

^6^ https://www.disgenet.org

^7^ https://string-db.org

^8^ https://davidbioinformatics.nih.gov/

^9^ https://www.rcsb.org/

## References

[1] Fan SJ Yang X Dong AJ. Diagnosis and treatment of pneumonia in calves. Hubei Ani Hus and Vet Med. (2011) 9:39–40. doi: 10.16733/j.cnki.issn1007-273x.2011.09.014

[2] Chen XJ Xiao XD. Pneumonia in calves: causes, prevention and case analysis. China Dairy Ind. (2022) 4:52–6.

[3] Manatibai S Kulizira H. Causes and prevention and treatment of pneumonia in calves. Chin Ani Hus. (2023) 11:97–8.

[4] Donkersgoed VJ Merrill KJ. Efficacy of tilmicosin for on-arrival treatment of bovine respiratory disease in backgrounded winter-placed feedlot calves. Bov Pra. (2013) 47:7–12.

[5] Saber APR Hoshyar BF Ajdari A. Control of clinical pneumonia in calves by antibiotic therapy. Eur J Exp Bio. (2013) 3.

[6] Wen C. Analysis of the effect of traditional Chinese medicine in the treatment of bovine pneumonia. J Chin Vet Med. (2022) 11:54–6.

[7] Liu WH Zhang TP Zhang XS. Clinical observation of tonifying lung soup in treating chronic persistent bronchial asthma. Chin Pra Med. (2018) 13:114–5. doi: 10.14163/j.cnki.11-5547/r.2018.12.068

[8] Wang CQ Tong HJ Wang L. Observation on the therapeutic effect of external application of spacer moxibustion combined with internal administration of Yiqi lung tonic soup plus reduction in the treatment of silicosis patients with lung qi deficiency. Chi Trad Chin Med Sci Tech. (2021) 28:604–6.

[9] Wen F Shu P. Network pharmacology of Astragalus-Codonopsis medicinal pairs in the treatment of gastric cancer. J Tradit Chin Med. (2021) 39:89-94+267-268. doi: 10.13193/j.issn.1673-7717.2021.02.024

[10] Wang J Wang H Liu P. Examination of the utility of Schisandra chinensis. J Pra Chin Med Int Med. (2017) 31:64–7. doi: 10.13729/j.issn.1671-7813.2017.12.22

[11] Du J Chen ZJ Chen XD Zhang JH Wang YJ Zhao TT. Inhibition of Glycyrrhiza polysaccharide on human cytochrome P450 46A1 *in vitro* and *in vivo*: implications in treating neurological diseases. Curr Drug Metab. (2024) 25:227–34. doi: 10.2174/0113892002305873240520072802, PMID: 38797896

[12] He LQ Cao JR Xie XL Zhang YY Zhang X Wang HB. Effects and mechanism of Qingke Pingchuan granules against influenza virus infection. Arch Virol. (2024) 169:130–15. doi: 10.1007/s00705-024-06053-z, PMID: 38807015

[13] Shin KC Oh DK. Biotransformation of platycosides, saponins from balloon flower root, into bioactive deglycosylated platycosides. Antioxidants. (2023) 12:327–7. doi: 10.3390/antiox12020327, PMID: 36829886 PMC9952785

[14] Wang XR Chang XW Zhi NN. Screening of expectorant, asthma-relieving, cough-relieving, and anti-inflammatory effective parts of Tongjiehuangdian from Anhui Province. Chin Mod Trad Med. (2024) 26:635–44. doi: 10.13313/j.issn.1673-4890.20231208001

[15] Li S Zhang B. Network pharmacology of traditional Chinese medicine: theory, methodology, and application. Chin Nat Med. (2013) 11:110–20. doi: 10.1016/s1875-5364(13)60037-0 23787177

[16] Zhang HM Liu SH Gao HJ. Progress of network pharmacology methodology of compounded traditional Chinese medicine. Chin Hosp Med Eval Analy. (2019) 19:1270-1273+1276. doi: 10.14009/j.issn.1672-2124.2019.10.035

[17] Zhang XS. Protective effect of Shengmusan on heat stress-induced liver injury in rats and its mechanis. GAU. (2022) 127. doi: 10.27025/d.cnki.ggsnu.2022.000063

[18] Li HM Xu K. Prevention and control of calf pneumonia by combining traditional Chinese and Western medicine. J An Hus Veter Med. (2022) 41:87–8.

[19] Zhang YL Liao QS Yang ZB. Technical points of calf pneumonia treatment. Con An Hus. (2015) 24:20–1.

[20] Mirza MA Mahmood S Hilles AR Ali A Khan MZ Zaidi SAA. Quercetin as a therapeutic product: evaluation of its pharmacological action and clinical applications—a review. Pharmaceuticals (Basel). (2023) 16:1631. doi: 10.3390/PH16111631, PMID: 38004496 PMC10674654

[21] Chen ZN Miao NY Zhu YC. Study on the effect of quercetin on the expression of TLR4, TRAF6, and IL-1β in mice with experimental tuberculosis. New Chin Med. (2019) 51:8–13. doi: 10.13457/j.cnki.jncm.2019.10.003

[22] Yang Y Chen Z Zhao X Xie H Du L Gao H. Mechanisms of Kaempferol in the treatment of diabetes: a comprehensive and latest review. Front Endocrinol (Lausanne). (2022) 13:990299. doi: 10.3389/fendo.2022.990299, PMID: 36157449 PMC9490412

[23] Pan J Meng L Li R Wang Z Yuan W Li Y. Naringenin protects against septic cardiomyopathy in mice by targeting HIF-1α. Biochem Biophys Res Commun. (2024) 704:149613. doi: 10.1016/j.bbrc.2024.149613, PMID: 38387325

[24] Li GW Ding JX Zhang YN Wang XY. The clinical application and pharmacological mechanism of Bu-Zhong-Yi-qi decoction for treating cancer-related fatigue: an overview. Biomed Pharmacother. (2022) 156:113969. doi: 10.1016/j.biopha.2022.113969, PMID: 36411646

[25] Zou CP Guo Y Zhang CW. Quercetin, an active ingredient in tonifying Zhongyiqi tang, enhances the sensitivity of lung adenocarcinoma drug-resistant cell line A549/DDP to cisplatin via the GSTP1-JAK-STAT pathway. J Guangzhou Univ Trad Chin Med. (2023) 40:2606–14. doi: 10.13359/j.cnki.gzxbtcm.2023.10.028

[26] Yang SJ Cui WW Wang MY Xing LM Wang Y Zhu PY. Bufei decoction alleviated bleomycin-induced idiopathic pulmonary fibrosis in mice by anti-inflammation. Evid Based Complement Alternat Med. (2020) 2020:7483278. doi: 10.1155/2020/7483278, PMID: 32963571 PMC7495219

[27] Pang L Han SY Jiao YN Jiang ST He XR Li PP. Bu Fei decoction attenuates the tumor-associated macrophage-stimulated proliferation, migration, invasion, and immunosuppression of non-small cell lung cancer, partially via IL-10 and PD-L1 regulation. Int J Oncol. (2017) 51:25–38. doi: 10.3892/ijo.2017.4014, PMID: 28534943 PMC5467788

[28] Al-Banna NA Cyprian F Albert MJ. Cytokine responses in campylobacteriosis: linking pathogenesis to immunity. Cytokine Growth Factor Rev. (2018) 41:75–87. doi: 10.1016/j.cytogfr.2018.03.005, PMID: 29550265

[29] Leal MC Casabona JC Puntel M Pitossi FJ. Interleukin-1β and tumor necrosis factor-α: reliable targets for protective therapies in Parkinson’s disease? Front Cell Neurosci. (2013) 7:53. doi: 10.3389/fncel.2013.00053 23641196 PMC3638129

[30] Rajput IR Li LY Xin X Wu BB Juan ZL Cui ZW. Effect of Saccharomyces boulardii and *Bacillus subtilis* B10 on intestinal ultrastructure modulation and mucosal immunity development mechanism in broiler chickens. Poult Sci. (2013) 92:956–65. doi: 10.3382/ps.2012-02845, PMID: 23472019

[31] Ehrenstein MR Cook HT Neuberger MS. Deficiency in serum immunoglobulin (Ig)M predisposes to development of IgG autoantibodies. J Exp Med. (2000) 191:1253–8. doi: 10.1084/jem.191.7.1253, PMID: 10748243 PMC2193170

[32] Li S Li J Liu Y Li C Zhang R Bao J. Effects of intermittent mild cold stimulation on mRNA expression of immunoglobulins, cytokines, and toll-like receptors in the small intestine of broilers. Animals (Basel). (2020) 10:1492. doi: 10.3390/ani10091492, PMID: 32846975 PMC7552237

[33] Wei H Ding L Wang X Yan Q Jiang C Hu C. (2021). Astragalus root extract improved average daily gain, immunity, antioxidant status and ruminal microbiota of early weaned yak calves. J Sci Food Agric. (2021) 101:82–90. doi: 10.1002/jsfa.10617, PMID: 32608134

[34] McNeil BK Renaud DL Steele MA Keunen AJ DeVries TJ. (2023). Effects of *Echinacea purpurea* supplementation on markers of immunity, health, intake, and growth of dairy calves. J Dairy Sci. (2023) 106:4949–65. doi: 10.3168/jds.2022-22862, PMID: 37268577

[35] Shen L Shen Y You L Zhang Y Su Z Peng G. Blood metabolomics reveals the therapeutic effect of Pueraria polysaccharide on calf diarrhea. BMC Vet Res. (2023) 19:98. doi: 10.1186/s12917-023-03662-9, PMID: 37516856 PMC10386334

[36] Ku JY Lee MJ Jung Y Choi HJ Park J. Changes in the gut microbiome due to diarrhea in neonatal Korean indigenous calves. Front Microbiol. (2025) 16:1511430. doi: 10.3389/fmicb.2025.1511430, PMID: 40109976 PMC11921620

[37] Fanelli A Cirilli M Lucente MS Zarea AAK Buonavoglia D Tempesta M. Fatal calf pneumonia outbreaks in Italian dairy herds involving mycoplasma bovis and other agents of BRD complex. Front Vet Sci. (2021) 8:742785. doi: 10.3389/fvets.2021.742785, PMID: 34568480 PMC8462733

[38] Kalaeva EA Alhamed M Kalaev VN. Prognostic value of hematological and biochemical parameters of mother and newborn calf for bronchopneumonia risk assessment in neonatal calve. Russ Agric Sci. (2021) 47:430–5. doi: 10.3103/s106836742104008x

[39] Lorenz I Earley B Gilmore J Hogan I Kennedy E More SJ. Calf health from birth to weaning. III. Housing and management of calf pneumonia. Ir Vet J. (2011) 64:14. doi: 10.1186/2046-0481-64-14, PMID: 22018053 PMC3220626

[40] Lv M Feng Y Zeng S Zhang Y Shen W Guan W. Quercetin and luteolin may be the new effective drugs for radiation pneumonitis: based on a systems pharmacology. Nat Prod Commun. (2022) 17. doi: 10.1177/1934578x221131126

[41] Zitzmann R Pfeffer M Söllner-Donat S Donat K. Risk factors for calf mortality influence the occurrence of antibodies against the pathogens of enzootic bronchopneumonia. Tierarztl Prax Ausg G Grosstiere Nutztiere. (2019) 47:151–65. doi: 10.1055/a-0899-1129, PMID: 31212341

[42] Shen J Zhu X Chen Y Li W Liu H Chu C. Bufei Decoction Improves Lung-Qi Deficiency Syndrome of Chronic Obstructive Pulmonary Disease in Rats by Regulating the Balance of Th17/Treg Cells [retracted in: Evid Based Complement Alternat Med. 2023 Dec 13;2023:9841968. doi: 10.1155/2023/9841968.]. Evid Based Complement Alternat Med. (2022) 2022:1459232. doi: 10.1155/2022/1459232 36034952 PMC9402293

